# Supplementary material for: The interactome of intact mitochondria by cross-linking mass spectrometry provides evidence for coexisting respiratory supercomplexes
Source: Mol Cell Proteomics. 2017 Dec 8;17(2):216–32. doi: 10.1074/mcp.RA117.000470 (PMC5795388; doi:10.1074/mcp.RA117.000470)
Supplement: Supplemental Data [file supp_RA117.000470_133922_0_supp_23978_fzffwf.zip › spectra_annotation/mito_DR_spectra_annotation/163-2-12-1-8-1.pdf]

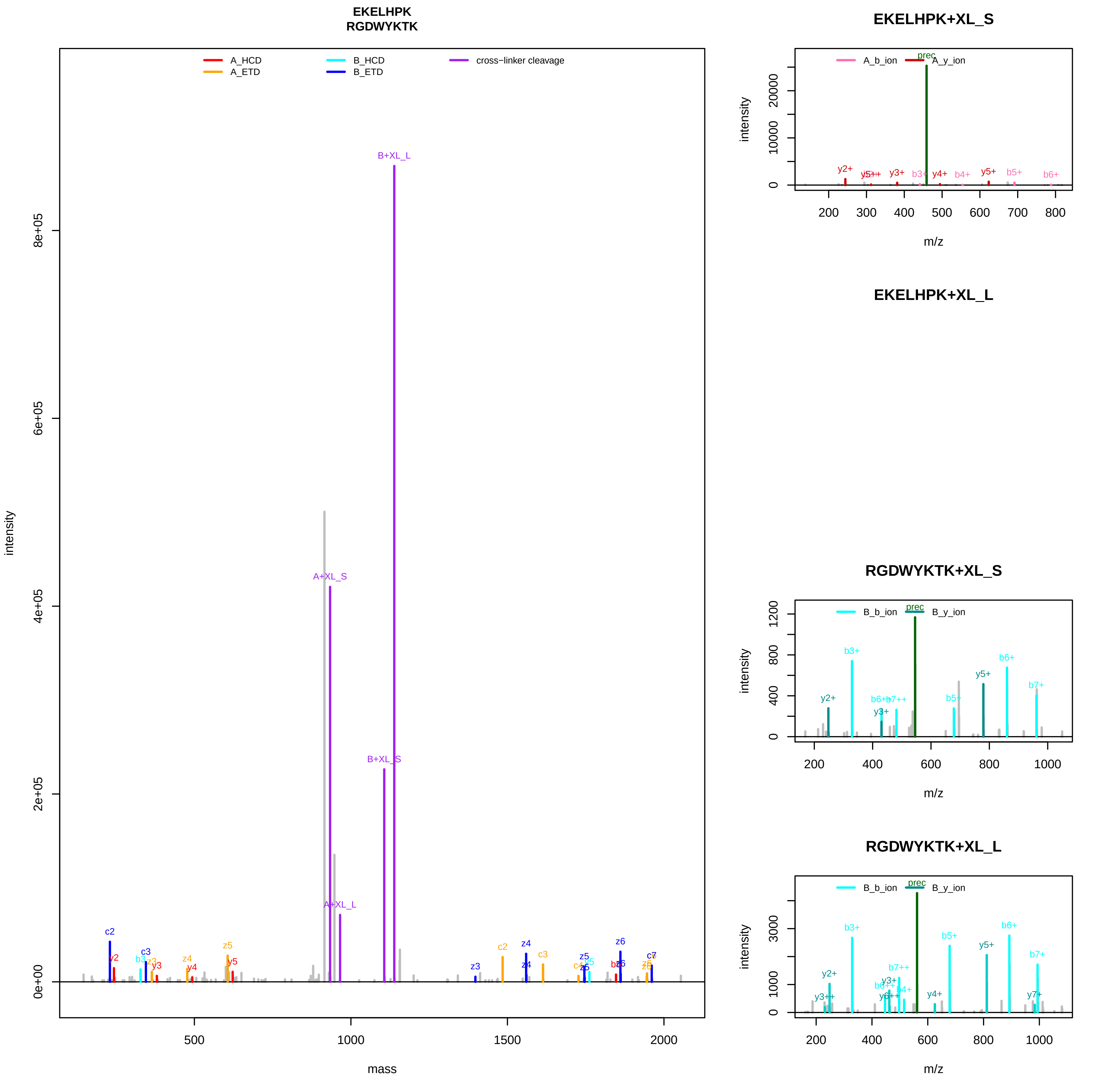
EKELHPK
RGDWYKTK
A_HCD
A_ETD
B_HCD
B_ETD
cross−linker cleavage
B+XL_L
A+XL_S
B+XL_S
A+XL_L
intensity
mass
0e+00
2e+05
4e+05
6e+05
8e+05
500
1000
1500
2000
c2
y2
b3
c3
z3
y3
z4
y4
z5
y5
z3
c2
z4
z4
c3
z5
y5
z6
c7
EKELHPK+XL_S
A_b_ion
A_y_ion
prec
y2+
y3+
b3+
y4+
b4+
y5+
b5+
b6+
m/z
EKELHPK+XL_L
RGDWYKTK+XL_S
B_b_ion
B_y_ion
b3+
y2+
y3+
b5+
y5+
b6+
b7+
RGDWYKTK+XL_L
b3+
y2+
y3++
b7++
b5+
y5+
b6+
b7+
y4+
y7+
